# Tissue mechanics drives regeneration of a mucociliated epidermis on the surface of *Xenopus* embryonic aggregates

**DOI:** 10.1038/s41467-020-14385-y

**Published:** 2020-01-31

**Authors:** Hye Young Kim, Timothy R. Jackson, Carsten Stuckenholz, Lance A. Davidson

**Affiliations:** 1 Department of Bioengineering, Swanson School of Engineering, University of Pittsburgh, Pittsburgh, PA 15213 USA; 2 Center for Vascular Research, Institute for Basic Science (IBS), Daejeon, Republic of Korea; 3 Essen BioScience, Ltd., Royston, SG8 5WY UK; 4 Department of Developmental Biology, School of Medicine, University of Pittsburgh, Pittsburgh, PA 15260 USA; 5 Department of Computational and Systems Biology, School of Medicine, University of Pittsburgh, Pittsburgh, PA 15260 USA

**Keywords:** Biophysical methods, Xenopus, Developmental biology, Embryology, Morphogenesis

## Abstract

Injury, surgery, and disease often disrupt tissues and it is the process of regeneration that aids the restoration of architecture and function. Regeneration can occur through multiple strategies including stem cell expansion, transdifferentiation, or proliferation of differentiated cells. We have identified a case of regeneration in Xenopus embryonic aggregates that restores a mucociliated epithelium from mesenchymal cells. Following disruption of embryonic tissue architecture and assembly of a compact mesenchymal aggregate, regeneration first restores an epithelium, transitioning from mesenchymal cells at the surface of the aggregate. Cells establish apico-basal polarity within 5 hours and a mucociliated epithelium within 24 hours. Regeneration coincides with nuclear translocation of the putative mechanotransducer YAP1 and a sharp increase in aggregate stiffness, and regeneration can be controlled by altering stiffness. We propose that regeneration of a mucociliated epithelium occurs in response to biophysical cues sensed by newly exposed cells on the surface of a disrupted mesenchymal tissue.

## Abstract (web summary)

The role of tissue mechanics in the regeneration of mucociliated epithelium in *Xenopus* is unclear. Here, the authors show that *Xenopus* ectoderm aggregates undergo epithelial-like phenotypic transition prior to differentiation of mucus-secreting goblet cells to enable regeneration.

## Introduction

Xenopus embryos develop a mucociliary epidermis that functions much like respiratory epithelia in mammals^1,2^. Goblet cell progenitors develop exclusively from the epithelial surface layer, whereas multiciliated and other accessory cells derive from deep layer cells^3–6^ following Notch-mediated patterning^7^. Once committed, multiciliated and accessory cell precursors intercalate between goblet cell progenitors to establish a fully functioning mucociliary epidermis^8^. Multiciliated and accessory cells have been extensively studied, yet the conditions driving goblet cell specification and the role of the mechanical microenvironment in establishing those conditions remain unclear.

Physical forces contribute to many cell fate decisions. For instance, during the first fate decision in the mouse embryo, polarized cell contractility generates asymmetric cell tension between superficial outer and inner deep cells, leading to separation of trophectoderm and inner cell mass^9^. Physical forces, or the structures they generate also appear to pattern feather follicles in avian skin^10^. In vitro studies suggest mechanics of the tissue microenvironment, physically defined by factors such as stiffness, geometry, and substrate topology, can direct stem cell lineage specification and renewal^11–13^; however, the contribution of such physicomechanical cues to embryonic cell specification and regeneration in vivo is poorly understood. This gap between in vivo and in vitro studies reflects a paucity of model systems where tissue mechanics, cell behaviors, and cell fate choices can be studied quantitatively. With access to single cells, tissues, and whole embryos, *Xenopus* development can serve as a tractable model system for quantitative investigations on the role of mechanical cues in embryonic cell specification and regeneration.

In this paper we describe regeneration of a mucociliated epidermis on the surface of embryonic aggregates and the role of tissue mechanics in converting mesenchymal cells into epithelial goblet cell precursors. Aggregates are assembled from cells isolated from the deep layer of gastrula stage *Xenopus* ectoderm tissues. We use these aggregates to investigate tissue mechanical properties during goblet cell regeneration and find that tissue compliance, a measure of tissue softness inversely related to stiffness, decreases during the early phase of epithelization and coincides with the nuclear translocation of the putative mechanotransducer YAP. To rule out simple correlation we separately increased and decreased compliance of the near-surface microenvironment. Using both small molecule inhibitors and mutant proteins we show that epithelialization can be blocked in high compliance or accelerated in low compliance environments. We show that mechanical cues alone can control regeneration of an embryonic mucociliary epithelium.

## Results

### Mesenchymal cells on surface transition to epithelial

**Fig. 1:**
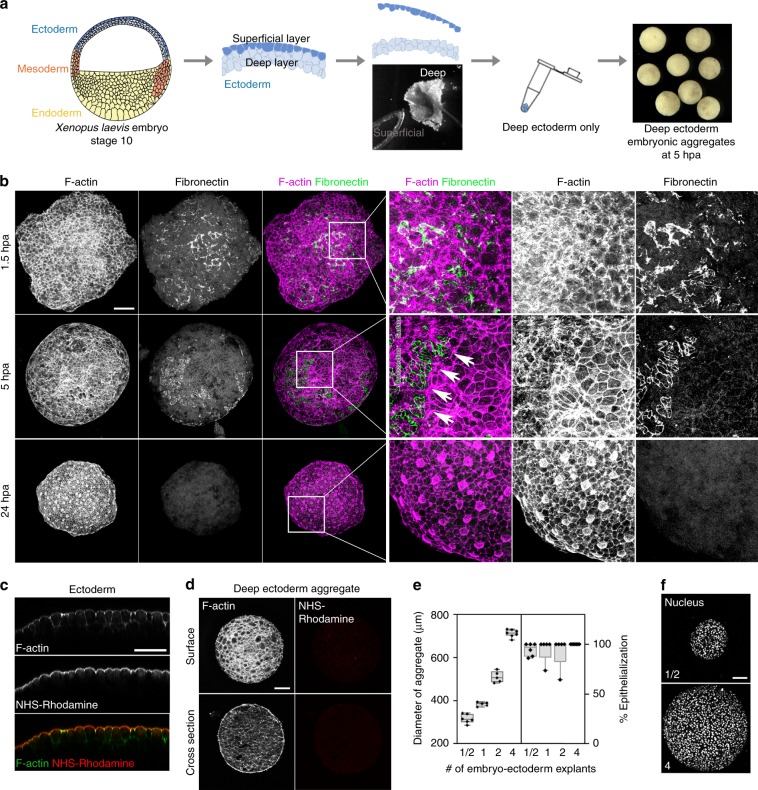
Surface cells of deep ectoderm aggregates undergo epithelial-like phenotypic transition. **a** Schematic of the assembly of deep ectoderm cell aggregates from early *Xenopus* embryo (Stage 10). **b** Surface F-actin and fibronectin (FN) from maximum intensity projections at 1.5, 5, and 24 h post aggregation (hpa). Three panels on the right are higher resolution views of the inset region (white box) in the third column. Arrows indicate margin of FN where dense circumapical F-actin suggests epithelial cell phenotype. Scale bar for aggregate images is 100 µm. **c** Transverse sectional view through the ectoderm of NHS-Rhodamine surface-labelled embryos. Scale bar, 50 µm. Rhodamine is restricted to the apical surface of outer epithelial cells. **d** Deep ectoderm aggregates generated from NHS-Rhodamine surface-labelled embryos. Scale bar, 100 µm. Lack of rhodamine indicates absence of contaminating epithelia. **e** Percent of epithelial cell phenotype found on the surface of different-sized deep ectoderm aggregates at 24 hpa. Aggregates assembled with varying amounts of embryo-ectoderm explants (1/2 explant, *n* = 6; 1 explant, *n* = 5; 2 explants, *n* = 5; 4 explants, *n* = 8). Box plot shows minimum, first quartile, median, third quartile, and maximum values. **f** Labeled nuclei shown in deep ectoderm aggregates from 1/2- and 4-embryo-ectoderm explant containing aggregates. Scale bar, 100 µm.

Deep mesenchymal cells isolated from embryonic ectoderm and shaped into aggregates undergo an unexpected, but profound transformation into an epithelial cell type. Embryonic cells isolated from deep layers of the *Xenopus laevis* embryo–ectoderm, i.e. cells immediately below the simple epithelium of the ectoderm, generate compact aggregates (Fig. 1a). Simple epithelia of the superficial cell layer assemble tight junctions^14^ and keratin intermediate filaments^15^, distinguishing them from deep mesenchymal cells. Differences in adhesion allow efficient separation of a superficial layer from deep layer cells by brief exposure to calcium–magnesium-free media (Fig. 1a). Isolated deep ectoderm cells transferred to a non-adherent centrifuge tube rapidly adhere to each other in <2 h to form a compact spherical aggregate. Immunostaining of F-actin and fibronectin (FN) show regions where surface cells extend F-actin rich protrusions and assemble fibronectin fibrils (Fig. 1b, 1.5 h post aggregation, hpa). However, by 5 hpa, clusters of cells on the aggregate surface are clear of FN fibrils and protrusions, and adopt distinctive epithelial-like shapes with sharp cell boundaries marked by dense F-actin cables (Fig. 1b, arrows). By 24 hpa, the entire surface develops into a mature epidermis devoid of FN fibrils, with multiciliated cells indicated by dense apical actin (Fig. 1b, Supplementary Fig. 1a). To rule out contamination by epithelial cells during microsurgery we surface labeled the outer cell layer of embryos used for making aggregates (Fig. 1c) and found no contaminating cells (Fig. 1d). Phenotypic transitions occurred across a range of aggregate sizes (Fig. 1e, f) from large (cells from four embryo–ectoderm explants) to small (cells from 1/2 of an embryo–ectoderm explant isolated from a single embryo). Thus, epithelial-like cells rapidly regenerate on the surface of a simple aggregate in the absence of externally provided factors.

**Fig. 2:**
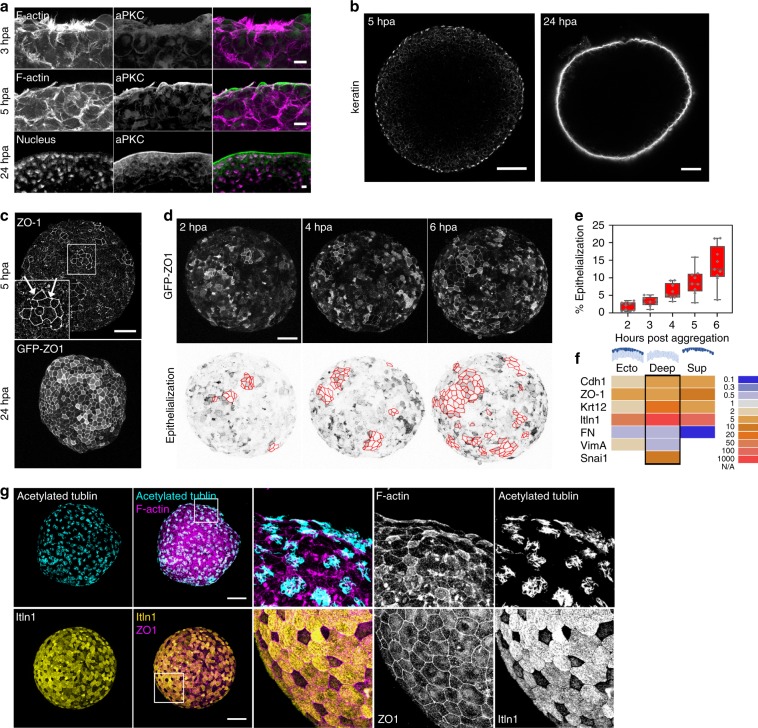
Epithelialization precedes differentiation of mucus-secreting goblet cells. **a** Apical polarity protein aPKC localizes on the apical surface of aggregates by 5 hpa. Scale bar, 10 µm. **b** Apical localization of epithelial cytoskeleton keratin is restricted to the outer surface of deep ectoderm aggregates. Cross sectional view of aggregates at 5 hpa (Scale bar, 100 µm) and 24 hpa (Scale bar, 50 µm). **c** Maximum intensity projection of epithelial tight junctional protein ZO-1 expression in aggregates at 5 hpa (top; immunofluorescence staining) and 24 hpa (bottom; GFP-ZO-1 expression). Epithelialized cells are marked by arrows (inset). Scale bar, 100 µm. **d** Representative frames from a time-lapse sequence of an aggregate expressing GFP-ZO-1. Top panel: maximum intensity projection of GFP-ZO-1 shows cells undergo epithelialization (outlined with red on lookup-table-inverted images in lower panel) on the surface of aggregates from 2 to 6 hpa. Scale bar, 100 µm. **e** Percentage of cells having undergone epithelialization increases over time (9 aggregates tracked from three clutches). Box plot shows minimum, first quartile, median, third quartile, and maximum values. **f** qPCR expression profiling in aggregates made of ectoderm, deep layer, or superficial layer cells. Expression of epithelial (Cdh1, ZO-1, Krt12, and Itln1) and mesenchymal (FN, VimA, and Snai1) genes are analyzed for CT-based fold changes from 3 hpa to 24 hpa. **g** At 24 hpa the deep ectoderm aggregate is covered by epithelial cells including differentiated mucus-secreting goblet cells (itln1) and radially intercalated multiciliated cells (acetylated tubulin). Scale bar, 100 μm.

Apico–basal polarity is established progressively in cells exposed on the surface of the aggregate. Cell membranes facing the external media accumulate aPKC, an early marker of apical polarity by 5 hpa; by 24 hpa, cell nuclei align in a pattern analogous to that seen in simple epithelia (Fig. 2a). By 5 hpa, epithelia-specific keratin intermediate filaments assemble along the apical surface; by 24 hpa, a mature epithelium forms with a dense keratin network (Fig. 2b). The epithelia-specific tight junction protein ZO-1 also appears on the outer surface of a subpopulation of cells by 5 hpa and covers the entire surface by 24 hpa (Fig. 2c). Live imaging of GFP-ZO1 at early stages reveals scattered puncta as well as circumapical junctions (Fig. 2c, d). Junctionally-localized GFP-ZO-1 appears on single cells and small groups as early as 2 hpa. The number of apically localized ZO-1 cells increases over time, accounting for 10 to 15% of the surface area by 6 hpa (Fig. 2d, e).

The phenotypic transition to epithelial-like cells coincides with changes in expression of epithelia- (Cdh1, ZO-1, Krt12, Itln1) and mesenchyme-specific genes (FN, VimA, and Snail) in ectoderm (both superficial and deep), deep ectoderm, and superficial ectoderm tissues between 3 and 24 hpa (Fig. 2f). Over this time-span, the aggregate increases expression of epithelial marker genes E-cadherin (Cdh1; 6-fold), ZO-1 (5-fold), and keratin (Krt12; >25-fold) and the goblet cell marker intelectin 1 (Itln1; >1500-fold) while continuing to express mesenchymal genes. The expression of mesenchymal genes in the aggregate reflects the continued presence of deep mesenchymal cells, even as the surface epithelializes (Fig. 2f). Together, these results indicate that surface cells of deep ectoderm aggregates transform into an epithelial cell type and regenerate an epithelium.

### New epithelium differentiates to mucociliary epidermis

How similar is this regenerated epithelial layer to the mature mucociliary epidermis of the embryo? *Xenopus* larval epidermis forms as deep progenitors of multiciliated cells, small secretory cells, and ionocytes radially intercalate into the outer layer formed by goblet cell precursors^6^. By 24 hpa, aggregates labeled with acetylated tubulin and F-actin reveal a pattern of multiciliated cells with dense apical actin cortex reminiscent of ciliated epithelium in similarly staged embryos (Fig. 2g). Furthermore, the ectoderm surface layer is dominated by mucus-secreting goblet cells marked by intelectin-1 (itln1 or Xeel; Fig. 2g). We further ruled out a source of goblet cells from Notch-dependent fate decisions that generate accessory cell types in vivo^7^ (Supplementary Fig. 1). Thus, the newly epithelialized surface of aggregates regenerates goblet cell precursors that are fully competent to differentiate into patterned larval epidermis analogous to epidermis observed in vivo.

### Epithelialization and YAP translocation as tissue stiffens

**Fig. 3:**
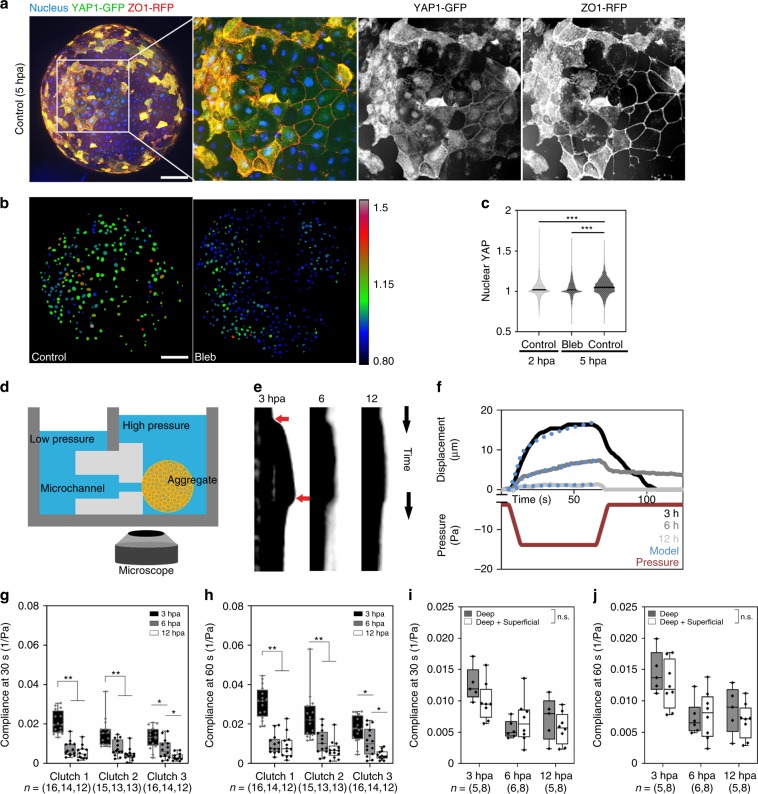
YAP nuclear translocation and tissue stiffening both coincide with epithelialization. **a** Maximum intensity projection of deep ectoderm aggregate expressing YAP1-GFP and ZO-1-RFP and stained for nuclei. Scale bar, 100 µm. **b** Color coded nuclear YAP ratios for control and blebbistatin treated deep ectoderm aggregates at 5 hpa. Scale bar, 100 µm. **c** Nuclear YAP ratios of 5 hpa aggregates (*n* = 2040 nuclei from 7 aggregates) are higher than blebbistatin-treated aggregates (3471 nuclei from 7 aggregates) and 2 hpa aggregates (2167 nuclei from 7 aggregates). Statistical analysis from one-way ANOVA followed by Tukey multiple comparison test (***P < 0.001). **d** Schematic of micro-aspirator used to measure tissue compliance of aggregates by adjusting pressure at the opening of the microchannel (see Methods). **e** Representative kymographs of tissue displacement over the length of representative micro-aspiration experiments at 3, 6, and 12 hpa. Red arrows indicate the time suction pressure is applied and then released. **f** Aspirated distances of an aggregates in (**e**) at 3 (black), 6 (dark gray), 12 hpa (light gray) relative to the pressure applied (red). The power-law model of creep compliance (blue dots) fit to this data. **g**, **h** Creep compliance at 30 and 60 s indicating steady-state from micro-aspiration at 3 (black), 6 (gray) and 12 hpa (white). 12 to 16 aggregates were measured for each time point and repeated over three clutches. **i**, **j** Creep compliance at 30 and 60 s from micro-aspiration of aggregates consisting of only deep mesenchymal cells (gray) or both deep mesenchymal and superficial epithelial cells (white) 3, 6, and 12 h post aggregation (one clutch, *n* = 5–8 aggregates each). **g**–**j** Box plots show minimum, first quartile, median, third quartile, and maximum values. Statistical analysis by Mann–Whitney U-test is shown as either not significant (n.s.) or by asterisk (*P < 0.05; **P < 0.01; ***P < 0.001).

What triggers de novo epithelialization and goblet cell differentiation of the surface-layer cells in aggregates? Unlike deep ectoderm cells in vivo, deep ectoderm cells on the surface of aggregates are unconstrained by adjacent epithelia. Emergence of apparently random epithelial patches across the surface (Fig. 2c, d) suggests that cells respond to locally varying mechanical conditions. To test if cells might be responding to changes in their mechanical microenvironment, we analyzed the localization of Yes-associated protein 1 (YAP), a transcription factor whose nuclear translocation in other cell types often correlates with changing mechanical conditions, e.g. stress fiber formation^16^, cell shape change and ECM rigidity^17^, and stretch^18^. YAP is found in the nucleus in cells on the surface of the aggregate (Fig. 3a) and its translocation to the nucleus depends on cell contractility (Fig. 3b). Nuclear levels of YAP increase in surface cells during the initial stages of epithelialization from 2 hpa to 5 hpa suggesting that surface cells experience changes in their mechanical environment as they establish apico-basal polarity (Fig. 3c). Since lower cell contractility reduced YAP nuclear translocation (Fig. 3b, c) we suspected that high levels of YAP translocation indicates an increase in tension at the aggregate surface.

To quantify mechanical changes in deep ectoderm aggregates, we measured the creep compliance, a function of tension and tissue stiffness, using micro-aspiration^19^. Application of a small negative pressure (−10.1 Pa) to a small patch on the surface of the aggregate reports compliance to a depth of ~125 µm (Fig. 3d–f). Deep ectoderm aggregates at 3, 6, and 12 hpa, representing phases before, during, and after the onset of epithelialization, respectively, exhibit significant and large decreases in compliance, i.e. increased stiffness, over the first 6 h, at the same time as the onset of epithelization; compliance continues to decrease but appears to stabilize by later phases of differentiation (Fig. 3g, h). Comparing the compliance of early aggregates (3 hpa) with or without superficial epithelia indicates that stiffness increases are not merely a consequence of epithelialization (Fig. 3i, j). Nuclear translocation of YAP together with phased reduction in compliance of the aggregates suggests that changes in the mechanical microenvironment promote surface epithelization in aggregates.

### Tissue mechanics controls epithelialization and regeneration

**Fig. 4:**
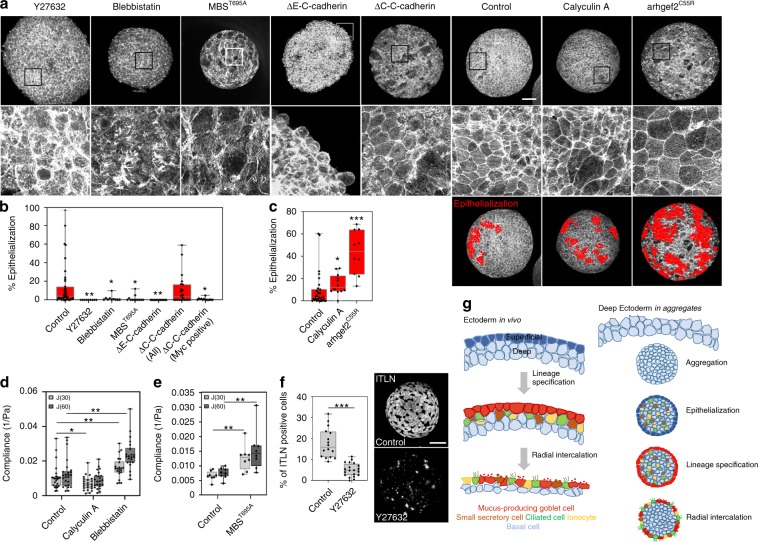
Contractility and adhesion regulate surface epithelialization and goblet cell specification. **a** Maximum intensity projection of F-actin stained aggregates at 5 hpa. Insets (boxes) in top row shown in lower row. Scale bar for all top row is 100 μm. **b** Epithelialization (Control, *n* = 39) is reduced after lowering contractility (Y27632, *n* = 9; Blebbistatin, *n* = 10; MBS^T695A^; *n* = 12) and altering cell–cell adhesion (ΔE-C-cadherin, *n* = 15 and ΔC-C-cadherin, *n* = 22). Analysis for mosaic ΔC-C-cadherin expression (anti-Myc positive) are shown in a separate bar. Kruskal–Wallis test, two-sided, (*P* = <0.0001 for Y27632, *P* = 0.015 for Blebbistatin, *P* = 0.001 for MBS^T695A^, *P* < 0.0001 for ΔE-C-cadherin, *P* = 0.147 for ΔC-C-cadherin, *P* = 0.001 for ΔC-C-cadherin (Myc positive)). **c** Epithelialization is increased after increasing contractility (control, *n* = 30; Calyculin A, *n* = 12; arhgef2^C55R^, *n* = 10). Red filled cells indicate epithelialized cells; surface areas are quantified in the graph. Significance of each treatment from the control is calculated using a Kruskal–Wallis H-test, two-sided; *P* = 0.046 for Calyculin A, *P* < 0.0001 for arhgef2. **d** Creep compliance at 30 and 60 s by micro-aspiration at 6 hpa after 4 h of small molecule inhibitor treatment. Data from four clutches were pooled with 22–25 aggregates per treatment. Statistical significance determined by Mann–Whitney U-test (*P < 0.05, **P < 0.01). (control, *n* = 25; Calyculin A, *n* = 23; Blebbistatin, *n* = 22). **e** Creep compliance for MBS^T695A^ expressing aggregates. Data from a single clutch with eight aggregates per treatment. Statistical significance determined by Mann–Whitney U-test (*P < 0.05, **P < 0.01). **f** Percentage of itln1 positive goblet cells quantified from two optical sections of 24 hpa in deep ectoderm aggregates (control, *n* = 8; Y27632, *n* = 10). Scale bar, 100 μm. Data from three clutches. Statistical analysis from unpaired *t*-test (two-tailed; *P* < 0.0001). **g** Schematic contrasting developmental sequence of native embryonic ectoderm with in vitro regeneration of surface goblet cells in deep ectoderm aggregates. Regenerated epithelium serves as a substrate for radial intercalation of multiple cell types including multiciliated cells, ionocytes, and small secretory cells. Regenerated epithelial cells differentiate into mucus secreting goblet cells. **b**–**f** Box plots show minimum, first quartile, median, third quartile, and maximum values.

To understand how mechanics might control epithelialization and goblet cell differentiation, we sought to test the roles of actomyosin contractility and cell–cell adhesion, key mediators of tissue mechanics in embryos^20^. To reduce contractility we expanded our earlier perturbations of cell contractility by incubating aggregates in either a Myosin II inhibitor, blebbistatin (100 μM), or a Rho-Kinase inhibitor, Y27632 (50 μM). To reduce cell–cell adhesion we expressed mutant forms of C-cadherin (cdh3), the major cadherin expressed within deep ectoderm cells at these stages. Mutant forms lacked either the cytoplasmic (ΔC-C-cadherin) or extracellular domains (ΔE-C-cadherin)^21^. By 5 hpa ~10% of the surface area of untreated aggregates adopt an epithelial phenotype (Fig. 4a, b) as quantified by the presence of circumapical F-actin, or ZO-1, along the boundary of the cells (Supplementary Fig. 2). Globally inhibiting contractility strongly reduces the epithelialized area, leaving the surface covered by F-actin rich protrusions (Fig. 4a) like that seen at earlier stages (compare to Fig. 1b). To confirm the specific effects of the small molecule inhibitors of contractility, we expressed a mutant myosin binding subunit (MBS, MYPT1, or formally PPP1R12A), MBS^T695A^, which is known to inhibit contractility and subsequently increase tissue compliance^22^. Expression of MBS^T695A^ blocked epithelialization (Fig. 4a, b). Reducing cell–cell adhesion by moderate overexpression of ΔE-C-cadherin also blocked epithelialization (Fig. 4a, b), as did moderate overexpression of ΔC-C-cadherin (Fig. 4a, b, ΔC-C-cadherin Myc positive); we note that wild-type cells (Myc negative) adjacent to mutant cells (ΔC-C-cadherin, Myc positive) retain the ability to transition to and become epithelial (Supplementary Fig. 3). The reduced incidence of surface epithelialization following reductions in contractility or adhesion suggests both are required to advance epithelization in the aggregates.

Since reduced contractility inhibited epithelialization, we wondered whether increasing contractility might accelerate epithelialization. To increase contractility, we incubated aggregates with Calyculin A (10 nM), a myosin light chain phosphatase inhibitor known to increase contractility^23,24^. Alternatively, we expressed a potent activator of myosin contractility, a constitutively active mutant form of arhgef2 (arhgef2^C55R^), a RhoA-specific guanine nucleotide exchange factor known to strongly lower tissue compliance^25^. Both treatments sharply increased epithelialization (1.6-fold by Calyculin A and 4.6-fold by arhgef2^C55R^; Fig. 4a, c). The effects of arhgef2^C55R^ can be attributed to cell contractility, since its effects on epithelialization can be completely abolished by incubation with Y27632 (Supplementary Fig. S4a and b). Micro-aspiration further confirms that Calyculin A reduces compliance (Fig. 4d), and that both Blebbistatin (Fig. 4d) and MBS^T695A^ (Fig. 4e) increase tissue compliance compared to control aggregates in concordance with their effects on actomyosin contractility. Furthermore, reduction of actomyosin contractility with Y27632 blocks goblet cell differentiation (Fig. 4f), indicating that tissue mechanics plays a role in both epithelization and differentiation; however, it remains unclear whether epithelialization is a prerequisite for goblet cell differentiation. In summary, our data shows that contractility and tissue compliance regulate the onset of epithelialization and regeneration of a mucociliary epithelia in embryonic aggregates (Fig. 4g).

## Discussion

Mesenchymal cell aggregates regenerate a superficial epithelial layer of goblet cell precursors in as little as 5 h. This phenotypic transition occurs in the absence of externally provided factors and is independent of endogenous patterning processes such as Notch-pathways that normally generate accessory cells in the deep ectoderm^26^. Unlike progenitor cells within stratified epithelia, or pseudostratified epithelia^27^, native deep ectoderm cells in *Xenopus* remain in the deep layer through tadpole stages^28^. By contrast, endogenous goblet cell precursors originate from, and are retained in the superficial layer of the ectoderm^6^. Thus, the programs driving epithelialization and regeneration of goblet cell precursors in aggregates appear to be distinct from endogenous developmental programs.

We are surprised that the genetic networks regulating goblet cell differentiation are distinct from the pathways generating multiciliated and accessory cells^29^. Our study suggests a novel goblet cell differentiation pathway that is Notch-independent and regulated by the mechanical microenvironment. It is interesting that multiciliated and accessory cells natively appear to undergo a form of single cell mesenchymal-to-epithelial transition^30^ as they intercalate between goblet cell precursors. The sensitivity of goblet cell precursor cells to mechanical cues may be responsible for pathological cases of basal cell differentiation where mucus-secreting goblet cells are over- or under-produced^31^.

The mechanical microenvironment surrounding progenitor cells can play a major role in determining their differentiation potential^11,32^ and in maintaining stem cell populations^32^. What mechanical cues drive epithelialization and regeneration of goblet cells? Formation of an aggregate involves cell–cell contact, increasing cell–cell adhesion, and cell–autonomous actomyosin contractility. While all cells generate cortical contractions and membrane extensions into their surroundings, cells embedded within aggregates are bounded on all sides by cell–cell adhesions, whereas cells on the surface of aggregates have two distinct surfaces: one that faces other cells and is bounded by cell–cell adhesion, and one that is open to the culture media and unconstrained by cell-cell adhesion. As the aggregate compacts, cell projections along the open surface contact neighboring cells in a manner similar to filopodial extensions that accompany compaction early in mouse embryogenesis^33,34^. We propose that as cell aggregates become tightly adhered, they decrease in tissue compliance (Fig. 3d–j) and trigger the de novo establishment of an apico–basal axis in surface cells, which subsequently assemble epithelial-specific junctions. The progressive polarization and assembly of apical junctions parallels critical events in the earliest stages of mammalian embryogenesis as the inner cell mass segregates into epithelial and deeper mesenchymal cell layers, leading to the first steps of cell fate specification^35^. Based on our findings, we speculate that strong apico–basal cues might trigger YAP translocation to the nucleus but cannot rule out the possibility that polarization is a consequence of YAP nuclear translocation.

How do cells on the surface of the aggregate sense compliance? Time-lapse confocal sequences reveal that mesenchymal cells in the aggregate are very active, moving onto and off the surface over the course of an hour. Such tumbling behavior may limit their ability to sense mechanical cues that initiate epithelialization. Reduced compliance, or increasing tissue solidity might reduce tumbling to produce a jammed state (e.g ref. ^36^) that stabilizes cell contacts and allows a persistent polarizing signal. What might cells be sensing? Cells may sense reduced compliance through protrusions that extend over their neighbors. This mechanism might rely on cell adhesion complexes that are stabilized and initiate signaling. Alternatively, cells may sense compliance changes via basolateral contacts with neighbors; such contacts could stabilize as compliance decreases, allowing cells to establish and maintain apico-basal polarity. With changes in adhesive contacts, or persistence of apical polarity, surface cells could initiate epithelial and goblet cell transcriptional programs.

*Xenopus* ectoderm has been used historically to dissect fundamental signaling and patterning networks of development and stem cell differentiation^37,38^. More recently, biomechanical studies of *Xenopus* ectoderm have demonstrated how mechanics plays a direct role in regulating intercalation^39^, cell division^40^, epiboly^41^, and directional beating of multiciliated cells^42,43^. Our findings suggest analysis of regeneration of *Xenopus* ectoderm can contribute to our understanding of the role of mechanics in regulating cell fate. Mechanical and biophysical approaches using *Xenopus* will allow the field to revisit critical questions of patterning and induction to understand how mechanics integrates with canonical patterning systems.

## Methods

### Embryos and embryonic cell aggregates

Embryos were obtained by standard methods; fertilized embryos were cultured in 1/3x modified Barth Solution (MBS)^44^ to the desired stage. To assemble the deep ectoderm aggregates, we first microsurgically isolated ectoderm explants from early gastrula embryos (Stage 10) in Danilchik’s For Amy (DFA; 44) medium supplemented with antibiotic and antimycotic (Sigma). Next, ectoderm explants were transferred into calcium-magnesium-free DFA which allowed separation of deep and superficial layers of the ectoderm explant. After discarding the superficial layer, freshly isolated deep ectoderm cells were transferred to a PCR tube using a pipette. To provide a non-adherent surface for aggregation, PCR tubes were coated with 1% BSA overnight at 4 °C. To confirm that our methods did not produce aggregates contaminated by epithelial cells from the superficial layer, the surface layer of whole embryos was labeled with NHS-Rhodamine (Thermo Scientific)^45^ and used to assemble deep ectoderm aggregates (Fig. 1c, d). Typically, 1–2 embryo–ectoderm explants were used to assemble a single aggregate except to make different sizes of aggregates (Fig. 1e, f). Deep ectoderm aggregates acquire a spherical shape within 2–3 h post aggregation (hpa), at which point they can be subjected to further procedures, including treatment with pharmacological inhibitors, measurement of compliance, and live imaging.

### Research animal use

Embryos used in this study were obtained from a colony of *Xenopus laevis* frogs maintained at the University of Pittsburgh (USA) under the care of the Division of Laboratory Animal Research according to IACUC Protocol #: 18022377 approved by the University of Pittsburgh Institutional Animal Care and Use Committee (PHS Assurance Number: D16-00118) as well as frogs maintained at the Institute for Basic Science (Republic of Korea) according to the protocol KAIST IACUC-(KA2017-22) / IBS IACUC (IBS 18-01).

### Microinjection of localization and overexpression constructs

To visualize or overexpress proteins, mRNAs encoding the desired proteins were transcribed in vitro and injected into one- or two-cell stage fertilized embryos. GFP-ZO-1 was constructed by moving the entire eGFP-human TJP1 fusion protein obtained from Addgene (Plasmid #30313; Addgene, Cambridge, MA) into pCS2+, mRFP-ZO-1 plasmid provided kindly by Dr. Ann Miller. To induce cell contractility, embryos were injected with arhgef2^C55R^ at ~87.5 pg per embryo^25^. To alter cell–cell adhesion, ΔE-C-cadherin (~1 ng per embryo) and ΔC-C-cadherin (~3.8 ng per embryo) were injected and ectopic gene expression was confirmed by both Myc staining and phenotype^21^. *Xenopus* Yap1.S (formerly YAP1) was amplified from a Stage 10 whole-embryo cDNA library and subcloned C-terminal to eGFP into a pCS2+ vector (InFusion Cloning, Takara, Mountain View, CA). The constitutive active form of a myosin binding subunit (MBS T695A) was obtained from the open reading frame of human protein phosphatase 1 regulatory subunit 12 A (PPP1R12A, MBS) and amplified from pGEX-PPP1R12A wild type (a gift from Anne Brunet, Addgene plasmid 31669;^46^) and subcloned into pCS2+. The threonine at position 695 was mutated to alanine by PCR sewing using overlapping primers. All constructs were verified by sequencing at the University of Pittsburgh Genomics Research Core.

### Histology and immunofluorescence

Aggregates were fixed with 4% paraformaldehyde and 0.25% glutaraldehyde in PBST (1x PBS with 0.1% Triton X-100) for 15 min at room temperature to visualize F-actin and fibronectin. For immunofluorescence of ZO-1, keratin, aPKC and Itln1, aggregates were fixed with ice cold Dent’s fix (4:1 of Methanol:DMSO). Fixed aggregates were washed for 30 minutes with PBST and blocked with 10% goat serum in PBST for 1 h prior to antibody staining. Primary antibodies for FN (4H2), aPKC (nPKCζ (C-20) sc-216; Santa Cruz), acetylated tubulin (clone 6-11B-1; Sigma), ZO-1 (Invitrogen), keratin (1h5; Developmental Studies Hybridoma Bank), Myc (9E10; Millipore), and Itln1 (gift from Dr. Eamon Dubaissi, 11770-1-AP; Proteintech) were used at 1:200 dilution and incubated overnight on a nutator at 4 °C. After washing, the samples were incubated with the appropriate secondary antibody at a 1:200 dilution overnight at 4 °C. F-actin and cell nuclei were visualized with BODIPY-FL–phallacidin (1:800) and either YO-PRO (1:10000, Invitrogen) or Hoechst 33342 (1:2000, Thermo Fisher), respectively. Cells were evaluated as having undergone epithelialization if they exhibited no F-actin protrusions and had strong circumapical F-actin, e.g. localization of F-actin to cell boundaries, which co-localize with ZO-1 (Supplementary Fig. 2).

For YAP nuclear localization studies, aggregates expressing YAP-GFP and ZO-1-RFP were fixed using 4% PFA in PBS for 15 min and stained with Hoechst. For analysis of the images, tiled images were stitched together using an ImageJ plugin^47^ and maximum projection images generated. The Hoechst channel was used to segment nuclei using a simple binary threshold. The YAP-GFP channel was used to segment and exclude cells not expressing fluorescent proteins. A custom macro quantified the mean nuclear intensity of YAP-GFP expression of each cell; the cytoplasmic intensity was obtained from an annular region surrounding each nuclei. YAP localization ratios (nuclear to cytoplasmic) are shown in Fig. 3b, c.

### Pharmacological inhibitors

To alter cell contractility, aggregates at 2 hpa were incubated with Y27632 (50 µM), Blebbistatin (100 µM), or Calyculin A (10 nM) until 5 hpa. Concentrations were chosen from prior studies in *Xenopus*^24,25,48–52^. All cytoskeletal inhibitors were purchased from Calbiochem (Millipore).

### Compliance measurements using micro-aspiration

To quantify tissue mechanics, aggregates were placed in the high-pressure reservoir of a dual-reservoir micro-aspirator apparatus^19,53^. Aggregates were gently compressed using hair tools against a 125 μm diameter channel cast across a PDMS block to form a seal across the opening of the channel (Fig. 3d). A pressure differential at the channel opening was controlled hydrostatically using a computer-controlled syringe pump. After calibrating the channel to obtain zero flow, a small baseline pressure of approximately −3.8 Pa was applied in order to initiate suction and seal the aggregate onto the channel opening. The tissue aggregate was allowed to relax for 5 min before the 125-s micro-aspiration protocol was initiated. At the start of the protocol, the aggregate was imaged at 1 frame per second to obtain an initial aspiration length for the tissue. After 5 frames, the syringe pump removed 800 μL of media from the low-pressure reservoir resulting in a −10-Pa suction pressure applied to the aggregate. After 60 s of −10 Pa suction, 800 μL of media was returned to the low-pressure reservoir to restore the baseline pressure. Imaging continued for an additional 60 s to confirm the aggregate was properly sealed onto the channel opening. When carrying out repeated measurements on the same sample, the aggregate was rotated to a different position. The tissue boundary during aspiration was tracked either manually or automatically with Canny-Deriche edge detection^54^. The aspirated length was measured and the data was fit to the power-law model for creep compliance, which has previously been shown to provide an adequate fit for embryonic tissue responses to stress-application^19^. Time-dependent compliance at 30 s (fast response) and 60 s (steady-state) is reported as the measurement of aggregate mechanical properties. Compliance calculations for each sample are independent of the magnitude of the pressure applied^19^. The suction pressure of −10 Pa was chosen in order to sufficiently displace aggregate tissues within the resolution of our imaging system without exceeding the critical pressure, thus ensuring measurements are representative of tissue viscoelasticity^55^.

### qPCR

To evaluate epithelial and mesenchymal gene expression in embryonic tissues, RNA was isolated using the Quick-RNA MiniPrep kit (Zymo Research) with 10 samples each of ectoderm, deep ectoderm aggregates, and superficial ectoderm aggregates at 3, 6, and 24 h post aggregation. RNA concentrations were measured using the NanoDrop and total RNA was used to create cDNA with the AccuScript High Fidelity 1st Strand cDNA Synthesis Kit (Agilent Technologies, Inc.). Forward and reverse primers (Supplementary Table 1) were designed for *Xenopus laevis* epithelial genes ZO-1, Cdh1, Itln1 and Krt12, mesenchymal genes FN, VimA and Snai1 and controls H4A. qPCR protocols were designed and carried out using the Bio-Rad iQ5 thermal cycler and PCR detection system. In all, 10 μL reaction volumes were prepared according to SsoAdvanced Universal SYBR Green Supermix (Bio-Rad) instruction manual. In all, 40 amplification cycles were carried out followed by a melting curve analysis to ensure product purity. Categorization of gene expression at 3 hpa was done using *C*_T_ values. Fold changes at 24 hpa were calculated using the Livak method, using H4 as the reference gene and 3 hpa as the calibrator^56^. Results are consistent across triplicate experiments with two replicates.

### Statistical analysis

Statistical differences of creep compliance among different time points post aggregation were calculated with ANOVA and planned repeated contrast. Stage dependence of compliance was measured over three clutches to account for clutch to clutch variation and potential staging errors. Statistical analysis of drug treatments was carried out using ANOVA and post-hoc analysis. ANOVA of drug treated aggregates showed data did not vary significantly by clutch (*P* > 0.05), so data was pooled from multiple clutches. Statistical tests of aggregate compliance or percentages of epithelialization were calculated using non-parametric methods using Mann–Whitney U-test (two cases) or Kruskal–Wallis H-test (multiple cases). Boxplots throughout paper describe the minimum, first quartile, median, third quartile, and maximum values. Statistical significance is shown as either not significant (n.s.) or by asterisk (*P < 0.05; **P < 0.01; ***P < 0.001). All statistical analyses and plots were carried out with statistical software (IBM SPSS, version 22 or Graphpad Prism 8.3.0). Fluorescence images shown are representative of the larger samples used for quantitation.

### Reporting summary

Further information on research design is available in the Nature Research Reporting Summary linked to this article.

## Supplementary information

Supplementary Information

Reporting Summary

## Data Availability

The data that support the findings of this study are available from the authors on reasonable request; see author contributions for specific data sets.

## Source data

Source Data
