# Supplementary material for: Tissue mechanics drives regeneration of a mucociliated epidermis on the surface of Xenopus embryonic aggregates
Source: Nat Commun. 2020 Jan 31;11:665. doi: 10.1038/s41467-020-14385-y (PMC6994656; doi:10.1038/s41467-020-14385-y)
Supplement: Supplementary file 1 — Supplementary Information [file 41467_2020_14385_MOESM1_ESM.pdf]

Supplementary Information for:

Tissue mechanics drives regeneration of a mucociliated epidermis on the surface. of Xenopus embryonic aggregates. Kim et al.

Supplementary Table

Gene	Alternate Name	GenBase Name	Accession	Forward Primer	Reverse Primer
Cdh1	E-Cadherin	Cdh1.S	XB-GENE-6464305	GCTGTTGTTGCTCTTACT	CGAGTCTCATCTTCTGGA
ZO-1	TJP-1	Tjp-1.L	XB-GENE-17332215	ATATCCAAGCAGTCAGAGA	TCATCTTCATCATCATCTTCC
Krt12		Krt12.S	XB-GENE-17333623	TTCCACATCACAATCATCTT	ACCACTTCTTCCACGATA
Itln1	Xeel	Itln1.L	XB-GENE-6256033	ACTGAGAGGGCTACACTTGCT	ATGTAACCACCTCCTCCAATGC
FN		Fn1.S	XB-GENE-865084	GGTGGAGGTGTGACAATT	TTGGTATCTCTGTGTAAACTGA
VimA		Vim.L	XB-GENE-866225	GCTAATCGCAACAATGATG	TTGAATAGTGTCCTGATAGTTAG
Snai1		Snai1.L	XB-GENE-865328	GGAGAGTCAGACAGTGTATA	CAAGAGGTGTGTAGTAAGC
H4		hist1h4a.L	XB-GENE-6493984	GACGCTGTCACCTACACCGAG	CGCCGAAGCCGTAGAGAGTG

Supplementary Table 1. Forward and reverse primers for *Xenopus laevis* epithelial genes ZO-1, Cdh1, Itln1 and Krt12, mesenchymal genes FN, VimA and Snai1 and control gene H4.

Supplementary Figures

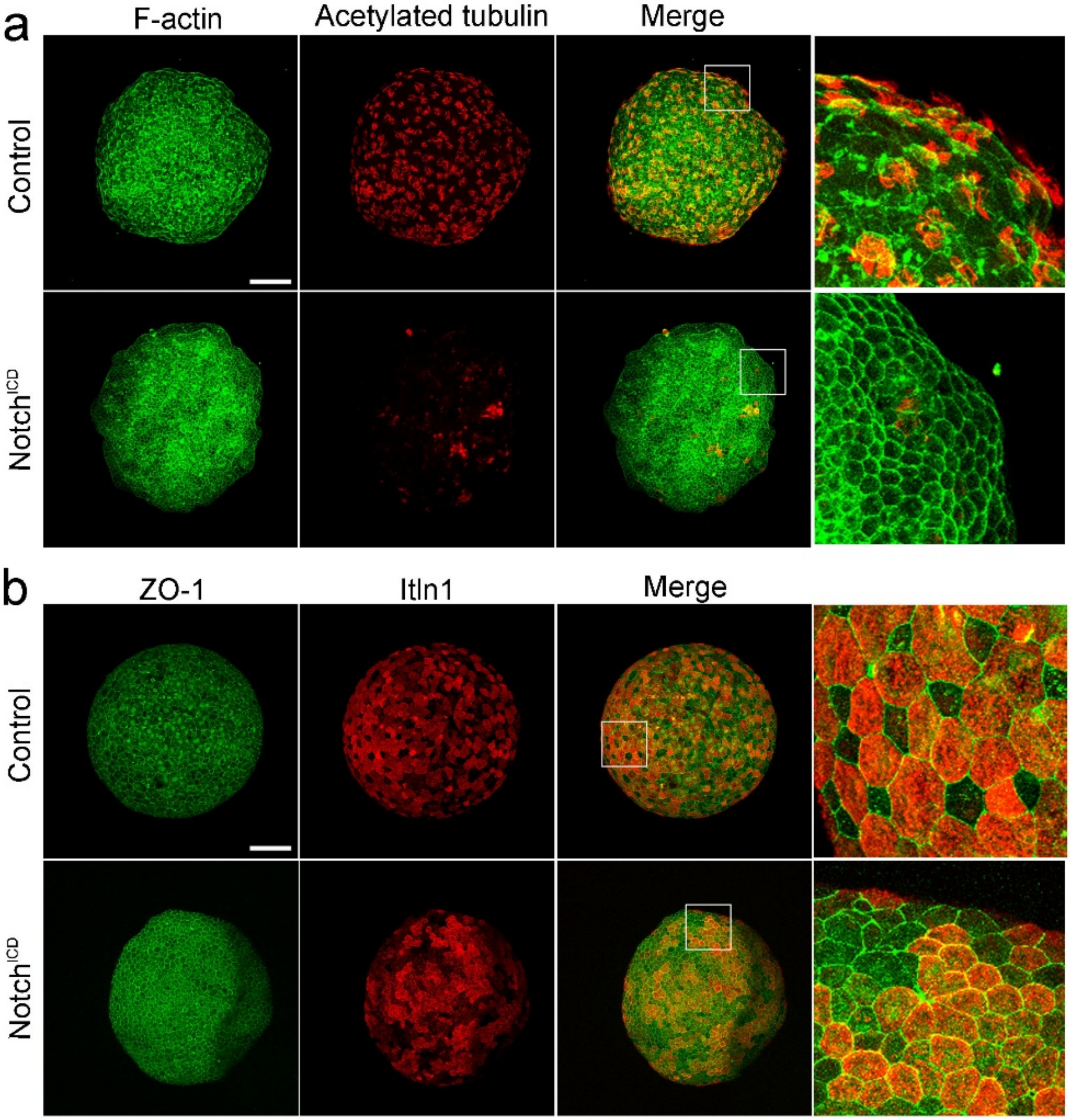

Supplementary Figure 1: Epithelialization and goblet cell specification is independent from Notch signaling.

a-b, Activation of Notch signaling by over-expression of the intracellular domain of $Notch^{ICD}$ inhibits the differentiation of multiciliated cells but does not inhibit regeneration of goblet cells in deep ectoderm aggregates (24 hpa). Inset regions of third columns (white boxes) are shown in fourth column. Scale bars are 100 µm for all image panels except insets.

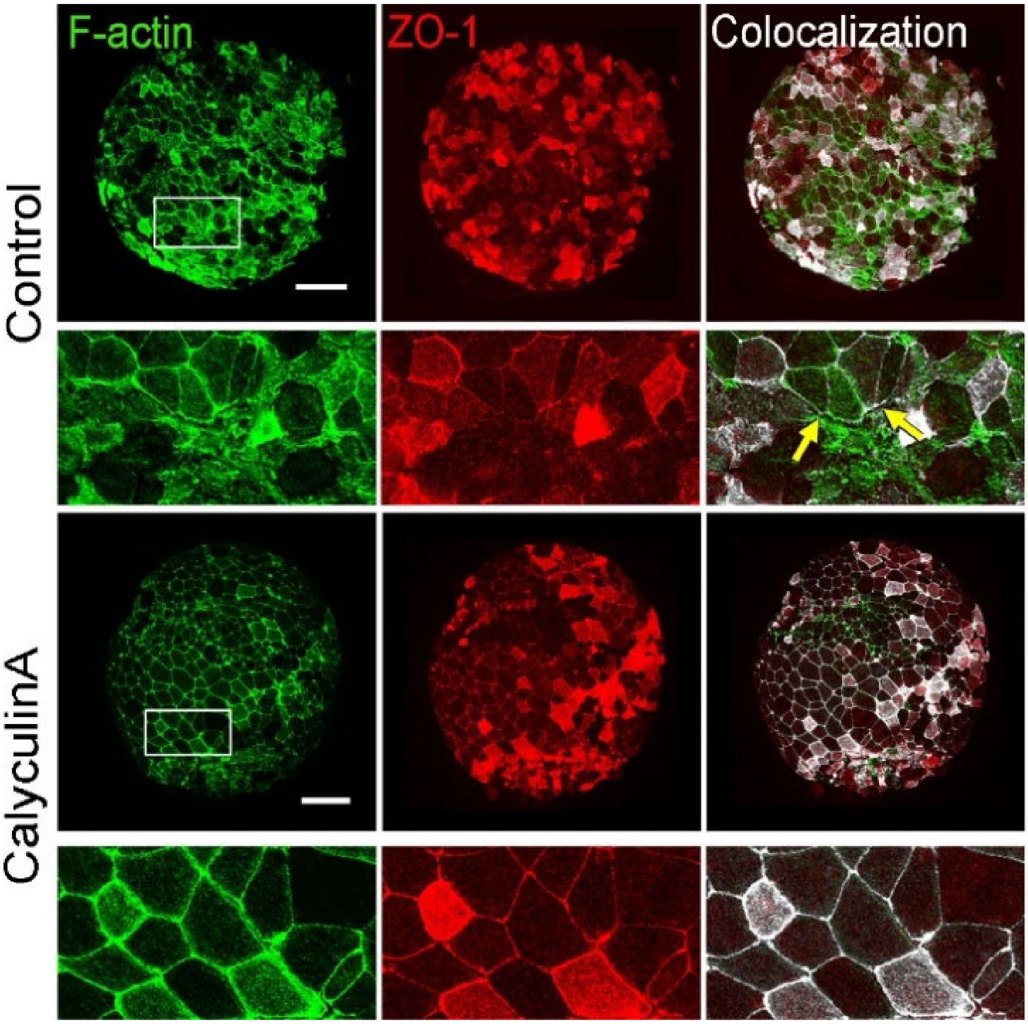

Supplementary Figure 2: F-actin and ZO-1 co-localize on the boundary of epithelial cells. Aggregates expressing ZO-1 RFP (red) and stained for F-actin (green) reveal co-localization of tight junctions and circumapical actin in epithelialized cells (white) in both control and Calyculin A treated aggregates. Inset regions from first and third rows (white boxes) are shown in second and fourth rows, respectively. Scale bars are 100 µm for all panels except insets.

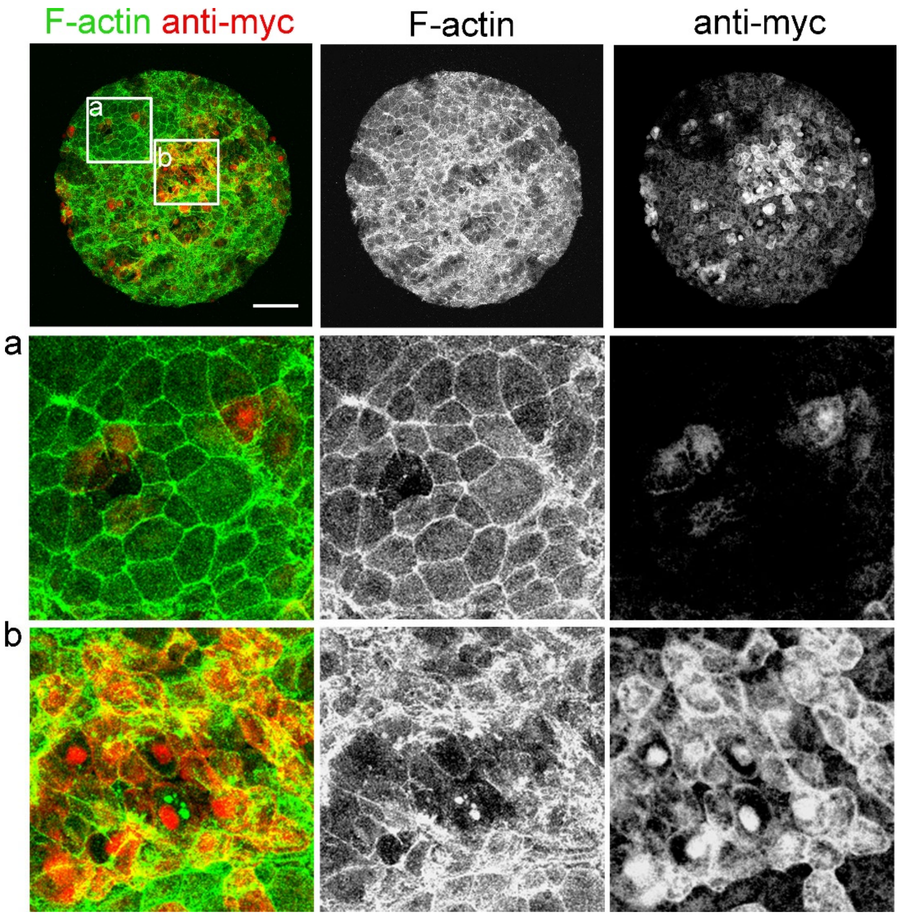

Supplementary Figure 3: ΔC-C-cadherin expressing cells are not able to transition to epithelial on the surface of deep ectoderm aggregates. F-actin and myc stained deep ectoderm aggregates at 5 hpa. Cells expressing myc-tagged ΔC-C-cadherin are detected by Anti-myc staining. Non-expressing cells epithelialize (a) while ΔC-C-cadherin expressing cells (yellow) do not (b) within same aggregate. Scale bar for whole aggregate (top row) is 100 µm.

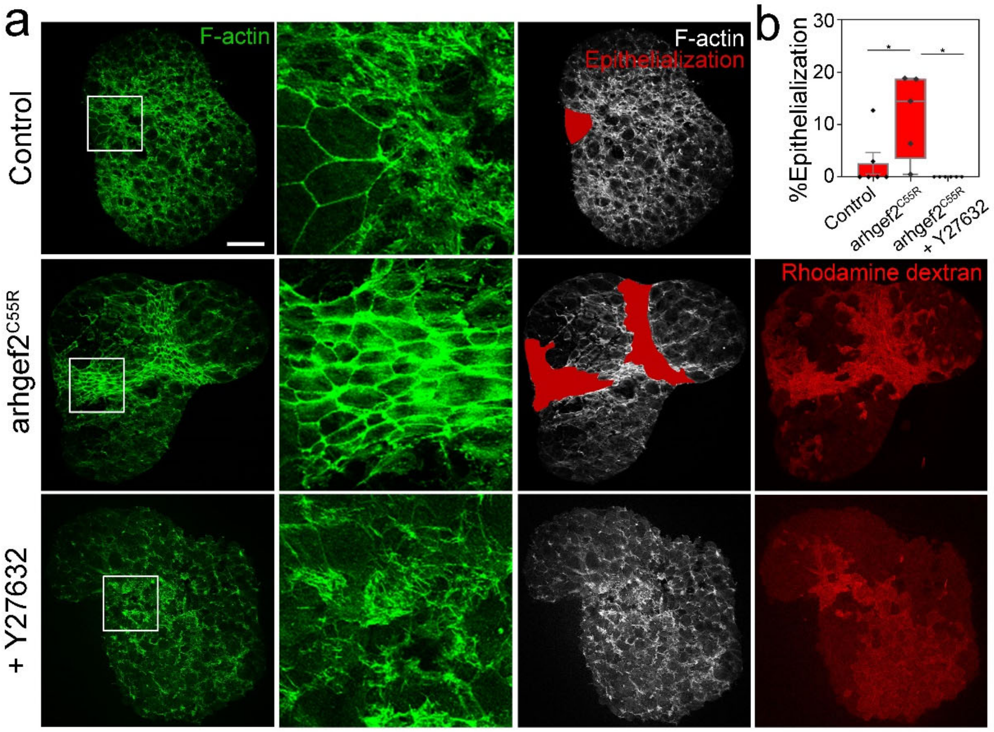

Supplementary Figure 4: ROCK inhibition blocks epithelialization driven by constitutive active Rho-GEF.

a. Representative maximum projection confocal images of F-actin labeled aggregates. Epithelialization proceeds in control 5 hpa aggregates is enhanced in arhgef2^{C55R} expressing aggregates. Incubation of arhgef2^{C55R} expressing aggregates in ROCK inhibitor Y27362 inhibits epithelialization. Inset regions in first column (white boxes) are shown in second column. Scale bar for all panels except the insets are 100 µm.

b. Percent of epithelialization from treatments shown in (a; controls, arhgef2^{C55R} , arhgef2^{C55R} + Y27362, n=6, 5, 6, respectively). (See Statistical Analysis in Methods for detailed description of statistical methods.)
