# Supplementary material for: Tissue mechanics drives regeneration of a mucociliated epidermis on the surface of Xenopus embryonic aggregates
Source: Nat Commun. 2020 Jan 31;11:665. doi: 10.1038/s41467-020-14385-y (PMC6994656; doi:10.1038/s41467-020-14385-y)
Supplement: Supplementary file 2 — Reporting Summary [file 41467_2020_14385_MOESM2_ESM.pdf]

nature research

Corresponding author(s): *Double-blind peer review submissions: write DBPR and your manuscript number here instead of author names.*

Last updated by author(s): *YYYY-MM-DD*

Reporting Summary

Nature Research wishes to improve the reproducibility of the work that we publish. This form provides structure for consistency and transparency in reporting. For further information on Nature Research policies, see Authors & Referees and the Editorial Policy Checklist .

Statistics

For all statistical analyses, confirm that the following items are present in the figure legend, table legend, main text, or Methods section.

n/a	Confirmed	
☐	☒	The exact sample size (*n*) for each experimental group/condition, given as a discrete number and unit of measurement
☐	☒	A statement on whether measurements were taken from distinct samples or whether the same sample was measured repeatedly
☐	☒	The statistical test(s) used AND whether they are one- or two-sided<br>*Only common tests should be described solely by name; describe more complex techniques in the Methods section.*
☐	☒	A description of all covariates tested
☐	☒	A description of any assumptions or corrections, such as tests of normality and adjustment for multiple comparisons
☐	☒	A full description of the statistical parameters including central tendency (e.g. means) or other basic estimates (e.g. regression coefficient) AND variation (e.g. standard deviation) or associated estimates of uncertainty (e.g. confidence intervals)
☐	☒	For null hypothesis testing, the test statistic (e.g. *F*, *t*, *r*) with confidence intervals, effect sizes, degrees of freedom and *P* value noted<br>*Give P values as exact values whenever suitable.*
☒	☐	For Bayesian analysis, information on the choice of priors and Markov chain Monte Carlo settings
☒	☐	For hierarchical and complex designs, identification of the appropriate level for tests and full reporting of outcomes
☒	☐	Estimates of effect sizes (e.g. Cohen's *d*, Pearson's *r*), indicating how they were calculated

Our web collection on statistics for biologists contains articles on many of the points above.

Software and code

Policy information about availability of computer code

Data collection	Microscope control and confocal images were collected using laboratory image acquisition software (Leica Microsystems, LASAF; Micromanager). Quantitative PCR was carried out with an integrated thermal cycler and detection system (BioRad, iQ5).
Data analysis	Images were analyzed with open source image analysis software (ImageJ) and data was analyzed for statistical significance using commercial software (IBM SPSS, version 22 or Graphpad Prism 8.3.0).

For manuscripts utilizing custom algorithms or software that are central to the research but not yet described in published literature, software must be made available to editors/reviewers. We strongly encourage code deposition in a community repository (e.g. GitHub). See the Nature Research guidelines for submitting code & software for further information.

Data

Policy information about availability of data

All manuscripts must include a data availability statement. This statement should provide the following information, where applicable:

- Accession codes, unique identifiers, or web links for publicly available datasets
- A list of figures that have associated raw data
- A description of any restrictions on data availability

The data that support the findings of this study are available from the authors on reasonable request; see author contributions for specific data sets.

Field-specific reporting

Please select the one below that is the best fit for your research. If you are not sure, read the appropriate sections before making your selection.

☒ Life sciences ☐ Behavioural & social sciences ☐ Ecological, evolutionary & environmental sciences

Life sciences study design

All studies must disclose on these points even when the disclosure is negative.

Sample size	In cases where epithelialization and biomechanical properties were assessed sample size was determined by prior experience to meet requirements for non-parametric statistical tests. ANOVA of compliance of drug treated aggregates showed data did not vary significantly by clutch (P > 0.05), so data was pooled from multiple clutches. Samples from experiments quantifying YAP localization data were pooled after analyses. Single data on YAP localization was pooled from multiple aggregates.
Data exclusions	No data was excluded from analysis.
Replication	Prior studies of tissue compliance demonstrated the need for tests to include 3 or more clutches (e.g. von Dassow, 2010) to limit the impact stage-to-stage variation. Imaging and mechanical testing were repeated for three biological replicates except where stated.
Randomization	Samples were separated into experimental groups by treatment.
Blinding	We did not blind the data source in the analysis pipeline. Image analysis and biomechanical testing studies were carried out using quantitative approaches and no sample sets were excluded from analysis.

Reporting for specific materials, systems and methods

We require information from authors about some types of materials, experimental systems and methods used in many studies. Here, indicate whether each material, system or method listed is relevant to your study. If you are not sure if a list item applies to your research, read the appropriate section before selecting a response.

Materials & experimental systems

n/a	Involved in the study	
☐	☒	Antibodies
☒	☐	Eukaryotic cell lines
☒	☐	Palaeontology
☐	☒	Animals and other organisms
☒	☐	Human research participants
☒	☐	Clinical data

Methods

n/a	Involved in the study	
☒	☐	ChIP-seq
☒	☐	Flow cytometry
☒	☐	MRI-based neuroimaging

Antibodies

Antibodies used	Primary antibodies for FN (4H2; Developmental Studies Hybridoma Bank), aPKC (nPKCζ (C-20) sc-216; Santa Cruz), acetylated tubulin (clone 6-11B-1; Sigma), ZO-1 (Invitrogen), keratin (1h5; Developmental Studies Hybridoma Bank), Myc (9E10; Millipore), and Itln1 (gift from Dr. Eamon Dubaissi, 11770-1-AP; Proteintech) were used. All secondaries were purchased from Jackson ImmunoLabs.
Validation	All antibodies had been previous validated in published studies.

Animals and other organisms

Policy information about studies involving animals; ARRIVE guidelines recommended for reporting animal research

Laboratory animals	Xenopus laevis frogs from our colony provided eggs (females) and testes (males).
Wild animals	No wild animals were used in the study.
Field-collected samples	No samples were collected from the field.
Ethics oversight	Embryos used in this study were obtained from a colony of Xenopus laevis frogs maintained at the University of Pittsburgh (USA) under the care of the Division of Laboratory Animal Research according to IACUC Protocol #: 18022377 approved by the University of Pittsburgh Institutional Animal Care and Use Committee (PHS Assurance Number: D16-00118) as well as frogs maintained at the Institute for Basic Science (Republic of Korea) according to the protocol KAIST IACUC-(KA2017-22) / IBS IACUC (IBS 18-01).

Note that full information on the approval of the study protocol must also be provided in the manuscript.
